# The infant disorganised attachment classification: “Patterning within the disturbance of coherence”

**DOI:** 10.1016/j.socscimed.2017.12.034

**Published:** 2018-03

**Authors:** Sophie Reijman, Sarah Foster, Robbie Duschinsky

**Affiliations:** a Primary Care Unit, Cambridge University, United Kingdom; b Department of Social Work & Communities, Northumbria University, United Kingdom

**Keywords:** United Kingdom, Classification, Psychology, Disorganised attachment, Infant mental health, Power/knowledge

## Abstract

Since its introduction by Main and Solomon in 1990, the infant disorganised attachment classification has functioned as a predictor of mental health in developmental psychology research. It has also been used by practitioners as an indicator of inadequate parenting and developmental risk, at times with greater confidence than research would support. Although attachment disorganisation takes many forms, it is generally understood to reflect a child's experience of being repeatedly alarmed by their parent's behaviour. In this paper we analyse how the infant disorganised attachment classification has been stabilised and interpreted, reporting results from archival study, ethnographic observations at four training institutes for coding disorganised attachment, interviews with researchers, certified coders and clinicians, and focus groups with child welfare practitioners. Our analysis points to the role of power/knowledge disjunctures in hindering communication between key groups: Main and Solomon and their readers; the oral culture of coders and the written culture of published papers; the research community and practitioners. We highlight how understandings of disorganised attachment have been magnetised by a simplified image of a child fearful of his or her own parent.

## Highlights

• Infant disorganised attachment is a classification used in mental health research.
• Divergent understandings of the classification have developed.
• We identify factors that have hindered communication about the classification.
• A simplified image of a baby afraid of its parent may have magnetised practitioners.

## 1 Introduction

An established and highly generative tradition of research and theory has explored how scientific and medical classificatory practices are constituted (Bowker et al., 2016, Hacking, 2004, Kendig, 2016), building from Foucault's pioneering work, for instance on societal images of mental illness as chaotic breakdown. Researchers have explored how classifications work, what they do, what relations they make, and with what consequences. Such inquiry attends to the practical activities scientists and clinicians enact (recording, describing, deducing, grouping, measuring, presuming, hazarding, talking past one another), not just the stabilised products of this work (empirical results, distributions of diagnoses, standardised protocols and systems of measurement, propositional knowledge, theories). Recently, one especially rich vein of research has been around the practical work of psychological diagnosis (e.g., Kendler et al., 2011, Moreira et al., 2008). Social scientists have engaged with debates regarding the legitimacy of psychological classifications, including whether they pick out enduring entities with exclusive boundaries, and their relative ‘constructedness’ or ‘independence’ from subjective judgement.

Infant “disorganised/disoriented attachment” (Main and Solomon, 1990), generally called “disorganised attachment”, is a classification made of infant-caregiver relationships in the Ainsworth Strange Situation. Though not a psychiatric diagnosis, it has been described as among the most influential assessments of infant mental health (Duschinsky, 2015, Lyons-Ruth and Jacobvitz, 2016). Disorganised attachment is generally regarded as the display of behaviours “lacking coherent pattern” (e.g., Schneider, 2014, p. 339). Mikulincer and Shaver (2016, p. 143) describe disorganised attachment as “random fluctuations” of behaviour. The mainstream account of the cause of disorganised attachment is that such chaotic breakdown “occurs when a child is simultaneously frightened of – or for – someone who they should be able to rely upon.” (National Institute for Health and Care Excellence, 2015, p. 20). These features - i.e., the randomness of the behaviours, and their common cause in fear of or for the caregiver - may be regarded as the orthodox account of disorganised attachment. As we shall see, this orthodox account, though right in some regards depending on exactly how terms are used, generally oversimplifies the phenomenon in important ways.

Among researchers, practitioners, and policy-makers, there has been “rapidly growing interest in disorganised attachment and subsequent child psychopathology” (Kochanska and Kim, 2013, p. 291). Infants classified as disorganised have an elevated risk of negative developmental outcomes; the most well-evidenced finding is a moderate association between the classification and later externalizing problems (e.g., aggression) across a variety of samples (Fearon et al., 2010, Sroufe et al., 2009), which is regarded by developmental scientists as comparatively strong among single predictors of behaviour problems. The National Institute for Health and Care Excellence (2015) have conducted a cost feasibility assessment for yearly screenings of all British infants for disorganised attachment as a developmental risk factor. Further indicating the currency of the construct, recent debates in the United States have examined whether and how disorganised attachment should be elevated to the status of a recognised clinical diagnosis (Zeanah and Lieberman, 2016). However, a consensus statement on disorganised attachment, published by leading researchers and clinicians in the area of child attachment (including Main and Solomon), attempts to qualify discussion of the classification (Granqvist et al., 2017, p .542):

The average effect size linking infant disorganized attachment with a particular caregiver to later behavior problems is small to moderate. In other words, a child assigned a disorganized classification is not necessarily expected to develop behavior problems. Additionally, when infants classified as disorganized do develop such problems, this may also be the result of a continuation of difficult life circumstances rather than solely an effect of early disorganized attachment.

Our study, begun in 2014, draws on historical and sociological analysis of the disorganised attachment construct to examine how it has been framed and with what consequences (see Duschinsky, 2015, Duschinsky et al., 2015, Duschinsky and Reijman, 2016, Duschinsky and Solomon, 2017, Reisz et al., 2017). Part of the context of our interest is signalled by remarks by Rutter et al. (2009, p. 532), that disorganisation “undoubtedly identifies behavioural features of considerable theoretical and clinical significance, but the meaning of the pattern remains rather unclear”. Likewise, Lyons-Ruth and Jacobvitz (2016) have argued that renewed efforts to clarify the meaning of disorganised attachment is critical for research and supportive work with infants and their families. A high-profile group of attachment researchers have also warned that work with the infant disorganised attachment classification “is limited by its reliance on a few scales that were not designed with psychometric modelling in mind” (Groh, Fearon, van IJzendoorn, Bakermans-Kranenburg and Roisman, 2017). In a watershed development in the wider context, the National Institute of Mental Health (2016) have removed the Ainsworth Strange Situation from their list of recommended procedures for publicly funded mental health research in the United States, polemically citing its debt to psychoanalysis and tendencies to “reify … theoretical claims” (p. 95) as reason to stop further funded research into attachment. Our study therefore occurs at a significant moment, potentially a turning-point, for the study of infant disorganised attachment.

### 1.1 Infant disorganised attachment: a background

Child-caregiver attachment has for several decades been a vibrant domain of research in developmental psychology and has had widespread influence in clinical, welfare, and forensic contexts. Foundational to the field is a laboratory-based assessment of infants' response to separation from and reunion with a familiar caregiver devised by Mary Ainsworth, the Strange Situation (Ainsworth et al., 1978). It was an early and influential form of a now common methodology: using structured observational research with populations who cannot be interviewed, treating behaviour as a window on participants' psychological state and history (Hollin and Pilnick, 2015).

Ainsworth and colleagues identified three patterns of response to the Strange Situation:

1) Most children showed distress on the departure of their caregiver, but were comforted on reunion and could return to play. This was in line with Bowlby's (1969) theory that a cue for danger, such as being left alone in a strange environment, would activate an infant's attachment response – their desire for the proximity and availability of a familiar adult – and that reunion and comforting would assuage this desire, allowing the child to turn their attention to exploration. Ainsworth's home observations found that the caregivers of these infants were responsive to their signals of distress, and she termed this a “secure” pattern of response in the Strange Situation.
2) A sizeable minority of infants did not show distress on separation, however, diverting their attention from the caregiver upon reunion, and not using the caregiver directly as a safe haven. Ainsworth's home observations found that the caregivers of these infants were relatively intrusive or dismissive of children's signals of distress; she theorised that the apparently unruffled behaviour of these infants in the laboratory masked the distress they could not show their caregiver. Several studies have found physiological patterns suggestive of stress in these babies during the Strange Situation, providing support for Ainsworth's hypothesis (e.g., Hill-Soderlund et al., 2008, Sroufe and Waters, 1977). She labelled this pattern of response “avoidant”.
3) A small number of infants displayed high levels of distress and desire for contact while also actively resisting comfort on reunion. They were unable to get settled and return to play following the separation. Ainsworth termed this a “resistant” pattern of infant-caregiver attachment. She observed that at home the caretakers of these infants gave their child reason to distrust their responsiveness, for instance through unpredictable attentiveness in many or most interactions when the child was distressed.

Researchers have found infant Strange Situation classifications to be associated with a wide variety of developmental outcomes including mental health, physical health, social competence, and moral reasoning (Sroufe et al., 2009). The Ainsworth patterns of attachment have been applied worldwide, and rates of security are generally consistent, except insofar as there are variations in the extent of adversity faced by families (Mesman, van IJzendoorn, & Sagi-Schwartz, 2016). They have been discussed by some attachment researchers as “natural kinds” that, adopting Plato's phrase, “carve nature at its joints” (Waters and Beauchaine, 2003, p. 417).

Yet Main and Solomon (1990) reported descriptions of infants who displayed behaviour suggestive of conflict or confusion which significantly disrupted an Ainsworth pattern of response (e.g., a child approaches the caregiver on reunion, but with her head sharply averted). Based on close analysis of 200 such cases, Main and Solomon introduced an additional “disorganised/disoriented” classification for the Strange Situation. However, they indicated that the disorganised classification was not of the same kind as the Ainsworth patterns: they advised an “underlying” secure, avoidant or resistant classification should be specified by coders where possible.

Main and Hesse (1990) theorised that one pathway to such conflicted or confused behaviour would occur when a distressed child wishes to approach their caregiver for comfort but also remembers times their caregiver's behaviour *alarmed* them, causing a desire to stay clear from the caregiver. Main and Hesse termed this pathway to disorganisation ‘fright without solution’. Children in this predicament were anticipated to be unable to direct their attention coherently either towards or away from the caregiver, resulting in conflict or confusion. Disorganised attachment was predicted, on this logic, not only in samples of maltreated infants, but also among children of parents who alarm their child for other reasons, for instance as a result of dissociative behaviours following trauma. The Main and Hesse hypothesis has received repeated support: a meta-analysis indicated that frightened or frightening caregiver behaviour during observations accounted for 13% of the variance in infant attachment disorganisation (Madigan et al., 2006). It was argued by Main and colleagues that in time most children would develop strategies to deal with such conflicts (either by aggressively controlling or caregiving and regulating their parent) and no longer display the forms of disorganised behaviour seen in infancy (Main and Cassidy, 1988). Nevertheless the term ‘disorganised’ has been extended to attachment assessments of older children and adolescents, sometimes used as a general and diffuse term for attachment pathology and incoherence with few substantive links with Main and Solomon's infant construct for coding the infant Strange Situation. For instance, the term ‘disorganised attachment’ was applied to adult or adolescent respondents who showed more than one salient profile on the researchers' self-report attachment questionnaire (Bifulco et al., 2016).

## 2 Methods

Our findings draw from three methodologies:

1) Archival study and literature review of the history and reception of Main and Solomon's publications. We were also provided access to coding notes for >300 cases from various samples seen in the Strange Situation, which were analysed using discourse analysis (Mottier, 2008), an approach concerned with what language is doing and how in reasoning about a topic.
2) Observations conducted at four training institutes for the coding of attachment disorganisation: with Elizabeth Carlson (July 2014, 2016), and with Judith Solomon (July 2015, July 2017). Training in the disorganised classification is given five or six days. We identified ourselves as researchers studying the training to participants from the start, and fully attended each training, transcribing verbatim as much as possible and taking additional ethnographic field notes. For our study, a particular value of ethnographic observation of learners of the coding system was that it took the opportunity to watch knowledge being formed as trained judgement, before it became tacit.
3) 32 semi-structured interviews, each around an hour and a quarter, conducted with researchers studying disorganised attachment, certified coders of the classification, and clinicians and social workers assessing and supporting children and families (with overlap between these groups). These included questions about how the individual first encountered the disorganised classification, what they understood its meaning to be, when and how they use it, and perceived strengths and limitations of the classification for the work that they do. Three focus groups were also conducted, each with between 17 and 26 British safeguarding professionals working with children and families. The interview and focus group data was again analysed using discourse analysis.

Our analysis was oriented by a concern with how the classification works in practice, beneath espoused discourse in the theory and methods sections of peer-review publications. We were keen to draw on the benefits of each methodology, and put them into dialogue (Maxwell et al., 2015). To achieve this we adopted a constant comparative methodology, which entailed the articulation of themes through a recursive movement between analysis of published narratives about disorganisation, and appraisal of kinds of convergent or divergent accounts in our archival, interview, and observational data.

Ethics approval for our study was granted by the Faculty of Health and Social Care at Northumbria University. Informed consent was obtained for data collection; verbal consent was obtained from individuals encountered during participant observation. We are grateful to Mary Main, Erik Hesse, Judith Solomon and Elizabeth Carlson for their work to support the research as gatekeepers, for instance through providing access to materials and trainings.

## 3 Results

All sources of data helped support the interpretation of the others, oriented by the overall aim of examining how the disorganised attachment construct has been framed and with what consequences. We distinguished three themes of analysis with bearing for this aim: the first mainly stemmed from our archival research and related to the authors' process of communicating the concept to readers; the second theme, especially apparent from the training institute observations, related to disparities in framing between published discussion of the classification and the oral culture of coders; the third, especially prominent in the interviews and focus groups, concerned the magnetic quality of the construct to an audience of practitioners working with children and families. Each theme is addressed in turn in the following sections.

### 3.1 Introducing the disorganised classification to readers

A first theme in our data, relevant to the framing of the construct and ensuing consequences, was the role of ambiguities of communication in shaping interpretations of the concept of disorganised attachment. Readers of Main and Solomon, 1986, Main and Solomon, 1990) chapters generally took from them the idea that disorganisation represents a unitary category of random behaviour caused by fear in relation to the caregiver – referred to above as the ‘orthodox account’ of disorganisation. However, in retrospect Main and Solomon have stated that they intended to draw attention to behaviours discrepant with the Ainsworth classifications suggestive of psychological disturbance, without implying that the behaviours were merely random or all reflect the same kind of fear. The authors have worked to correct assumptions about the meaning of their classification as its popularity has grown with practitioner audiences (e.g., Main et al., 2011, Solomon et al., 2017). They acknowledge that aspects of their writing misdirected readers, and that in hindsight further qualifications and clarifications would have been beneficial to defend against hasty readings of their ideas.

There were a number of reasons for complications in the communication between the authors of the classification and their readers. One fundamental factor was and remains confusion between everyday and scientific language. This is a common issue for psychological discourse, where terms like ‘depression’, ‘anxiety’ and ‘trauma’ operate across discursive registers, with relatively different meanings between everyday and specialist language. Confusions between ordinary language and technical psychological uses of the term ‘disorganised’ have an especially long and varied history (Leeper, 1948). In ordinary language, ‘disorganisation’ means undifferentiated chaos: “to break up the organic connection of; to throw into confusion or disorder” (Oxford English Dictionary online). A use of the term within clinical language closer to this ordinary language meaning is in the diagnosis of ‘disorganised schizophrenia’, one of the five subtypes of schizophrenia, characterised by speech and emotions perceived as meaningless, confused and disordered. This was not the intended meaning of the term by Main and Solomon, who defined disorganisation, specifically, as “an observed contradiction in movement pattern, corresponding to an inferred contradiction in intention or plan” (Main and Solomon, 1990, p. 133). This usage laminates two different levels of analysis: 1) marked conflict or confusion in infants' *visible* behaviour with their caregiver, and 2) an *inferred* disruption in the coordination of infant behaviour, attention and affect into a plan for achieving or maintaining the availability of their familiar caregiver when distressed (the attachment response).

Another complication in the communication between the authors and their readers resulted from the implied meaning of gathering the “disorganised” behaviours under a single classification. Main and Solomon (1990) closely analysed 100 recordings of infants from “low-risk samples” and 100 recordings from “high-risk samples” (including maltreated infants, infants of traumatised parents, and from families experiencing chaos and poverty) and proposed certain infant behaviours to be indicative of a disorganised attachment response. They clustered the identified behaviours into seven indices based on their morphology:

I. Sequential displays of contradictory behaviour;
II. Simultaneous display of contradictory behaviour;
III. Undirected, misdirected or incomplete movements;
IV. Stereotypies, mistimed movements and anomalous postures;
V. Freezing or stilling;
VI. Display of apprehension of the caregiver;
VII. Overt signs of disorientation.

As Main and Solomon acknowledged, behaviours pertaining to indices I-V were discussed by Hinde (1966) and Bowlby (1969), who termed them ‘conflict behaviours’ as they were observed in animals in situations when countervailing motivations would likely be present. Main and Solomon introduced two further kinds of behaviour based on their analysis of the recordings: apprehension directed towards the caregiver (VI), and disorientation or confusion on reunion or in proximity with the caregiver (VII). Unlike the mostly discrete Ainsworth patterns, Main and Solomon found that infants who showed apprehension or disorientation *also* tended to show conflict behaviours (though not necessarily vice versa). This led them to regard the phenomena as highly related, even if not necessarily identical in meaning. Consequently the non-exhaustive, varying behavioural indicators were grouped together as a single classification (see Duschinsky, 2015).

To place this decision in context, in a field of empirical inquiry grounded on Ainsworth's Strange Situation and her patterns of attachment, categorical classifications were the accepted currency; to a large extent they formed a horizon of how data could readily be conceptualised and discussed at the time of the introduction of the disorganised classification. No psychometric analysis was performed to examine whether the behaviours in the Main and Solomon indices represented a category. And at the time, Main wondered in print whether use of a single, encompassing category to capture these infant behaviours would be misleading (Main and Cassidy, 1988). However, the creation of a new category was generally taken by readers, on the assumption that this was the position of Main and Solomon, as implying that the behaviours reflected a unitary process. Only recently has this assumption received interest in theoretical discussions of attachment, in part on the tide of a wider concern across psychological science since the early 2000s to replace categories with dimensions in the interests of psychometric precision and statistical power (Fraley and Spieker, 2003, Groh et al., 2017; for discussion of this wider tide see e.g., Kendler et al., 2011).

In taking stock of the reception of Main and Solomon's work, it is important to highlight that the disorganised classification was formed *not* as the operationalisation of a construct, but as the categorical grouping of a list of pre-existing anomalies. Following Main and Hesse (1990), these have largely been considered the result of alarm elicited by the infant's experiences of their caregiver. Yet as the authors have subsequently attempted to clarify for their readers, with limited success, there can be a variety of causes of alarm (see e.g., Hesse and Main, 2006). A doctoral project under Main and Hesse's supervision explored this matter empirically. In a multiple regression with several parenting predictors -a including threatening and frightened behaviour - only dissociative parenting behaviour, which was also the most prevalent, predicted disorganised attachment (Abrams et al., 2006). Hesse and Main (2006, p. 335) advise that it would be “a worthwhile endeavor for developmental psychopathology” to further study different caregiving contexts and “compare these to the forms of disorganised behaviour exhibited by their infants”.

A decade later this call is yet to be answered. There are a number of reasons for this. The disorganised classification is usable as a predictor of child mental health within a progressive theory-driven research programme, and the field's priority has understandably been to secure evidence of prediction. Additionally, psychometric work is inadequately professionally rewarded for psychological researchers. However, based on the published literature and our interviews with researchers, we also suspect the operation of a mutually reinforcing relationship between lack of conceptual clarity regarding the disorganisation construct among many attachment researchers and difficulty conducting psychometric research. One factor contributing to this cycle has been the need for samples of adequate size for psychometric research, but this has not been the only factor in play since a number of such samples have long been available now.

An early influence on this cycle was the curtailment of certain complexities in Main and Solomon's published chapters. Notable disparities between Main and Solomon's understanding of and questions about their construct in the period of its introduction and the relatively simplified and closed account available for readers are visible in three differences between the original version of Main and Solomon's 1990 chapter and the published text. According to Solomon, writing in retrospect, this curtailment had three reasons: the authors were intending to make a complex phenomenon more intelligible to their audience; they were trying to reduce the length of already over-long chapters; and these complexities by no means had a place within available theory to make them salient at the time (Solomon et al., 2017).

First, in the original manuscript Main and Solomon note that in their 200 tapes the large majority of children displaying Index VI and Index VII behaviours were from maltreatment or very high-risk samples. However, this observation was cut from the already lengthy published chapter; without a theoretical framework to make this observation interpretable, it appeared as a mere detail. Yet from the vantage of the present, this difference in preponderance of behaviours between infants from different samples seen by Main and Solomon is likely to have been a meaningful finding. It is supported by the landmark Minnesota Longitudinal Study in which, with an exploratory aim, Index VI-VII were also identified as potentially distinct from Index I-V: infants classified in the Strange Situation as disorganised who displayed Index I-V behaviours (and *no* Index VI or VII behaviours) had shown less neonatal affect regulation than infants who did display Index VI and/or VII behaviours, suggesting that the former behaviours may be predisposed by neurological difficulties or other individual-level differences (Padrón et al., 2014).

Second, in a similar vein, Main and Solomon originally offered speculations regarding potential subclassifications of disorganised attachment, for instance distinguishing “apprehensive” disorganisation from other subclassifications and predicting, in a footnote, that this may have distinct sequelae. However speculations about different forms of disorganised attachment were cut from the published version. (Due to an oversight in the authors' revision process, the subclassifications of disorganised attachment nonetheless surface in Table 2, but have never been mentioned in any subsequent published text, nor were they mentioned in the training of coders.) Attachment research as a scientific research programme depends upon ready inter-coder agreement about what is being seen, and Main and Solomon did not attempt to examine whether agreement on such behaviours was possible. Viewed as subclassifications of disorganisation, it may have been assumed that it would be harder to achieve reliability between coders on finer details. However, in retrospect it is perhaps not self-evident that achieving coder agreement in, for instance, the distinction between overtly apprehensive versus non-apprehensive infant behaviours towards their caregiver would have been more difficult than weighting different behaviours in determining assignment of the disorganised attachment classification as a whole.

A third difference between the original manuscript and the published chapter was the illustrations. In both versions, pen drawings were presented of infants showing disorganised behaviour. The drawings were tracings of the film negatives of recordings of infants in the Strange Situation. In the original manuscript, the illustrations show a variety of the behaviours. In the published version, the only illustrations are depictions of infants whose facial expressions convey either terror or crumpled misery. These drawings of apprehension on reunion are better as drawings, more lifelike than the ones selected for removal, but their selection for the published version of the text may also have in part been shaped by theory. In a later chapter of the same book as Main and Solomon's introduction of the classification, Main and Hesse (1990) proposed that a child classified as disorganised may experience ‘fright without solution’ in wishing to seek their caregiver for comfort, but being alarmed in some way by their experiences with the caregiver. Illustrations of visible infant fear on reunion with the caregiver therefore served as a powerful encapsulation and expression of the process theorised to varying degrees to underlie disorganised behaviours.

The particular influence of visual representation for the interpretation of both new classificatory systems and the emotional state of others is well documented (Coopmans et al., 2014), and the choice of illustrations by Main and Solomon may well have been important for how readers imagined the disorganised infant. The image of fear on reunion with a parent is central, for example, to discussion of disorganisation in textbooks for psychology students (e.g. Parke and Clarke-Stewart, 2011), and guidance provided for social workers and child safeguarding practitioners (e.g., Shemmings and Shemmings, 2011) as well as for clinical psychologists by the British Psychological Association (BPS, 2017). The assumption that the different forms of disorganised behaviour had the ultimate meaning of fear of the parent was also present in the accounts offered by many of the clinicians and social workers when we asked in interview and focus groups what was entailed by infant disorganised attachment.

### 3.2 The oral culture of coding

A second theme in our data was the importance of the oral culture of coders, and how this differed from published discussions on the topic of disorganisation. Among non-coders, and based on the published record, disorganisation is regarded as comparatively rare: overall, 15% of infant-caregiver relationships are classified as disorganised in community samples. While classification rates tend to be higher in high-risk samples (34% in families facing economic adversity, 77% in maltreatment samples when the Main and Solomon indices were used; van IJzendoorn, Schuengel and Bakermans-Kranenburg, 1999), these are harder to recruit in large numbers. However, the *behaviours* are far from rare. Would-be coders at the training institutes talked of their surprise on learning that in samples where there is any risk factor facing the families (which applies to the majority of studies funded over recent decades) most infants show at least some readily visible behaviour characterised in the Main and Solomon indices. Indeed, the primary deliberation associated with coding is in judging whether the *extent* of disorganisation warrants or does not warrant assignment to the overall category; there is basic written guidance for making this judgement, and in practice is a skill achieved by observing experts and attempting to calibrate with them. Many trainee coders referred to this process as an attempt at “mind-merging” with the expert coders.

Furthermore, based on ideas circulating in the published literature, ‘disorganisation’ was expected by new trainees to be visible as a chaotic breakdown of behaviour. Yet in fact coders were instructed to seek a certain quality of order in making the classification for cases: in Elizabeth Carlson's training institute, attendees were advised to attend to “repetition and patterning within the disturbance of coherence”. Following the Main and Hesse (1990) hypothesis that disorganisation in some way signifies historical experience of alarm in relation to the caregiver, would-be coders were taught to consider *whether* and *in what way* infant fear is present, with an emphasis on the (absence of) function and meaning of individual behaviours or sequences of behaviour in the child's relationship with their caregiver and the extent to which these disrupted the pattern of infant response. Coders were told that they could more confidently interpret attachment disorganisation if an infant displayed “themes within the disturbance” than in the face of a smattering of apparently unrelated behaviours from among the Main and Solomon indices.

This reasoned hunt for (lack of) order in making a disorganised attachment classification was also observed in many of the decisions of other experienced coders, including in the coding notes of Mary Main and Erik Hesse. To give an illustration from one case: a coder was not certain whether a child falling abruptly to the floor at father's gentle touch on the first reunion was disorientation (Index VII) or an infant's expectable poor motor control. The coder justified her leap to a disorganised classification by citing the infant's tension movements (Index IV) and combined crying and avoidance (Index II) when picked up by father on the second reunion. Though quite different from one another, and possibly weak evidence taken alone, the combined logic of these behaviours in their context was understood by the coder as indicating the baby's worry about touch.

Aspiring coders are required to develop a high level of tacit skill in distinguishing forms of behaviour within the Main and Solomon indices, and weighing their relative significance for disruption of the attachment response. In light of the basic written guidance and the need for “mind-merging” with the expert coder in order to achieve this, training in the coding of disorganisation entails a continuous dialogue between trainees and the expert coder as trainees ask directed questions, based on taped examples of disorganised behaviours, to try and lay bare the parameters by which the coder assesses these behaviours. The sometimes blurry transitions between organized and disorganised behaviours, the boundaries and overlap between the 7 main disorganised categories, and the extent to which fear is visible or inferred, are open and crucial topics of discussion. However, the dialogues and considerations that are an intrinsic part of coders' training and practical knowledge are mostly absent from discussions around disorganisation in the published literature. This may in part be because high impact-publications require robust, replicable associations, and because the time-consuming coding work is generally passed to research assistants who are not necessarily involved in manuscript development. In any case, debates around disorganised attachment in the public domain show a lack of transparency in the intricacies and workings of the classification; these have largely remained “under the radar”, in the oral culture of coders.

### 3.3 Practice audiences

A third theme in our data highlighted the attractiveness of the disorganised attachment concept for practitioners. In general, classification is an important part of how professionals engage with clients' problems (Hacking, 2004). Our focus groups with British safeguarding professionals indicated that appeal to individual differences in attachment offers a way for practitioners working with children and families to “emphasise the importance of relationships, emotion, and the impact of early experience”, a relatively different focus from the dominant cognitive and behavioural approaches within therapeutic practice, at least in Britain. Yet, in contrast to psychoanalysis and in common with cognitive and behavioural approaches, attachment classifications are regarded as “grounded in science”. The Strange Situation classifications reflect palpable behaviour, and the credibility of the coding system makes the behaviour seem legible to practitioner audiences as a window into a child's mental health. In this light, attachment classifications hold out the prospect of greater certainties compared to other forms of knowledge available for handling the urgent complexities of child safeguarding assessments.

To give one example, Wilkins (2012) recommends the disorganised attachment classification to social workers and child welfare professionals as a way to cut through the particularities of potential maltreatment cases, to see through culture and class to the needs of a child and their likely future outcomes, yet without suggesting that professionals need to undergo training in reliably coding the classification. In a resource-strapped context, the appeal of the disorganised classification as a window on the child's history of caregiving is understandably appealing; and safeguarding professionals report that it has helped them effectively identify risk to children (Wilkins, 2017). However, Granqvist et al. (2017) flag that to date there has been no empirical evaluation of this risk-identification strategy, and that false positives are likely given the multiple pathways to the behaviours identified in the Main and Solomon indices.

During the July 2015 training institute, Judith Solomon urged recognition among trainee coders of the problems with the deployment of disorganised attachment in social work assessments, especially by practitioners without training in coding the classification:

There's premature use of disorganised attachment as a diagnostic label for assessment of risk, and it's dangerous. For example, those of you who work with children in the foster care system will know that when they are visiting their parents, the children's behaviour at reunion is frequently disorganised. “Ah-ha!” Social workers say. But is this because of the repeated separations? Or because they are frightened of the parent? Or because they are alarmed by the situation and their parent in it? No one knows; it has not been teased apart. It is a big quandary for social workers, and I don't think they know it; they get caught up in this image of disorganisation which floats free from the actual referent and how we operationalise the construct.

Yet as well as advocacy for the disorganised classification in child maltreatment assessments by Wilkins and others, there has also been pushback from some clinicians and social workers against the operationalisation of disorganisation as a single encompassing category. Even before Main and Solomon's publications, clinicians had been familiar with different behaviours now grouped under the disorganised classification (e.g., Fraiberg, 1982). Since Melanie Klein and Anna Freud, articulating varying forms of conflict in a child's relation to the caregiver has been among the primal therapeutic tasks in psychodynamic work with children. In child therapeutic practice, disoriented/dissociated responses by an infant to a parent are usually treated as quite different in antecedents and significance to depression, or to overtly fearful responses to a parent.

The wider issue of the disjuncture between generalised systems of classification and the specific, fine-grained knowledge of practitioners has also been documented by other scholars (e.g., Moreira et al., 2008), and was a major preoccupation of Bowlby's own writings (e.g., Bowlby, 1988). Generalised systems of classification and practitioner perspectives forms of knowledge with different kinds of epistemological standing, specificity, and concerns. In our case, the difficulties of linkage between research on disorganised attachment and professional experience has increased use by practitioners of models of attachment that simply bypass the disorganised classification in clinical formulation – most notably appeal to an over-extended reconfiguration of the concept of “Reactive Attachment Disorder” (Woolgar and Baldock, 2015). In recent years, the disjuncture between practitioner experience and research on disorganisation has produced a growing body of published discussion and debate. For example in the *Handbook of Attachment*, the field's key reference text, Lyons-Ruth and Jacobvitz (2016) have stated that bridging practitioner knowledge and attachment research on disorganisation is a ‘priority’ for the field. Yet their proposal to disaggregate the disorganised classification and validate those parts that are especially predictive of risk into a recognised diagnostic category as a way to achieve this bridge has been controversial.

## 4 Conclusions

Pickering (2015) has described how our machines for knowing, whether telescopes or classifications, are used and tuned until a point of ‘interactive stabilisation’. When this occurs, these machines for knowing, by informing us in some serviceable way about the world, take on a certain density. The disorganised classification has been tuned to one such point of interactive stabilisation, however, Pickering urges recognition that multiple usable points of interactive stabilisation will likely exist; which of these points is “best” will depend on what we want from the instrument. The tuning of the disorganised attachment classification has clear advantages for aggregative work within a research programme for a field of inquiry dependent upon time-intensive observational research. However, with the accumulation of large datasets, some have observed that dependence solely on an encompassing categorical approach to the phenomenon may also have some drawbacks. As we have seen, Hesse and Main (2006) have called on researchers to examine the correlates of different forms of disorganisation to understand more about the phenomenon. Such inquiry may also help bring closer research and practice interests in child attachment. A comparison could be drawn in this regard with the issues facing major depression, operationalised as a unitary category with largely interchangeable indicators – though in the higher-profile case of major depression the question of aggregation or specification has been more widely recognised and researched (Fried et al., 2016). There are also some similarities with debates between those who find an undifferentiated diagnosis of “chronic lower back pain” usable, well aligned with patient report and helpful for meta-analytic work, and those who feel that a higher resolution tool identifying subgroupings or stratification of risk would, if evidence of reliability and validity were secured, have advantages for resource allocation (Foster et al., 2014).

As in these cases, there may be points of contingency in the way that the disorganised attachment construct has stabilised, and the possibilities that stem from this. To give an example of how this may be relevant: where studies have found dramatically divergent results between high and low-risk samples (e.g., Haltigan and Roisman, 2015), the notion that this could be a consequence of *varying forms* of disorganisation differentially predominating in the respective samples has not apparently been considered, but remains a testable hypothesis. As discussed above, varying forms of disorganisation are an active topic of discussion among coders of the classification, but not in the published literature. We hope there are coders who may write and publish about what it is they are seeing and coding in order to narrow the gap between the oral and printed traditions, which may stimulate research questions that were previously unaddressed. Another point of contingency is, we suspect, be the extent of the difficulties of communication between researchers and their wider reader constituencies. As well as a contribution to the study of classificatory practices within psychology, we hope that the account presented here may help to clarify and begin to address some of these difficulties of communication about the infant disorganised attachment classification.
